# Bedside Evaluation of the Functional Organization of the Auditory Cortex in Patients with Disorders of Consciousness

**DOI:** 10.1371/journal.pone.0146788

**Published:** 2016-01-20

**Authors:** Julie Henriques, Lionel Pazart, Lyudmila Grigoryeva, Emelyne Muzard, Yvan Beaussant, Emmanuel Haffen, Thierry Moulin, Régis Aubry, Juan-Pablo Ortega, Damien Gabriel

**Affiliations:** 1 Laboratoire de Mathématiques de Besançon, Besançon, France; 2 Cegos Deployment, Besançon, France; 3 INSERM CIC 1431 Centre d’Investigation Clinique en Innovation Technologique, CHU de Besançon, Besançon, France; 4 EA 481 Laboratoire de Neurosciences de Besançon, Besançon, France; 5 Service de neurologie, CHU de Besançon, Besançon, France; 6 Département douleur soins palliatifs, CHU de Besançon, Besançon, France; 7 Service de Psychiatrie de l’adulte, CHU de Besançon, Besançon, France; 8 Centre National de la Recherche Scientifique (CNRS), Paris, France; University of British Columbia, CANADA

## Abstract

To measure the level of residual cognitive function in patients with disorders of consciousness, the use of electrophysiological and neuroimaging protocols of increasing complexity is recommended. This work presents an EEG-based method capable of assessing at an individual level the integrity of the auditory cortex at the bedside of patients and can be seen as the first cortical stage of this hierarchical approach. The method is based on two features: first, the possibility of automatically detecting the presence of a N100 wave and second, in showing evidence of frequency processing in the auditory cortex with a machine learning based classification of the EEG signals associated with different frequencies and auditory stimulation modalities. In the control group of twelve healthy volunteers, cortical frequency processing was clearly demonstrated. EEG recordings from two patients with disorders of consciousness showed evidence of partially preserved cortical processing in the first patient and none in the second patient. From these results, it appears that the classification method presented here reliably detects signal differences in the encoding of frequencies and is a useful tool in the evaluation of the integrity of the auditory cortex. Even though the classification method presented in this work was designed for patients with disorders of consciousness, it can also be applied to other pathological populations.

## Introduction

The appropriate management of patients with disorders of consciousness (DOCs), such as comatose patients, or patients with an unresponsive wakefulness syndrome (UWS, previously known as vegetative state) or patients in a minimally conscious state (MCS), highly depends on their level of residual cognition.

Since the behavioral assessment of residual cognitive functions can be extremely difficult, the use of functional neuroimaging is now frequently recommended. Several authors advocate defining the degree and extent of preserved cognitive functions through electrophysiological and neuroimaging protocols of increasing complexity [1,2]. Surprisingly, whereas the more complex stages of this hierarchical approach have been largely investigated, the first cortical responses emerging from the primary sensory cortices have suffered from a real lack of interest.

This is especially the case in the auditory domain, the easiest modality to assess in patients with DOCs. Apart from usual clinical investigations of the auditory pathway with brainstem auditory evoked responses, the integrity of the primary auditory cortex (AC), an essential gateway to higher and more specialized cortical areas [3,4], has not been fully explored. So far, neuroimaging and electrophysiological studies on patients with DOCs only measured whether there was a response from the AC [5,6] but did not evaluate its frequency-processing abilities, a key organizational feature of this structure. Yet it is of major importance because it is not possible to comprehend speech unless it has been perceived and encoded correctly in the brain. One reason that may explain this lack of interest is that the tonotopic organization of the AC is extremely difficult to measure with non invasive methods because of the small size of the auditory cortical fields and the interindividual variability in the anatomy of the Heschl’s gyrus [7]. It is only with the emergence of high field (≥3 Tesla) functional MRI that more consistent tonotopic maps of the AC have been described [8,9]. However, because of the low availability of a scanner, the cost of an fMRI exam, and the necessity of transferring the patient to the machine, fMRI assessments of cortical tonotopy can hardly be generalized to pathological populations. In comparison, a less expensive, and potentially transportable to the patient’s bedside, method such as electroencephalography (EEG) associated with source reconstruction algorithms only managed to demonstrate the tonotopic organization of the AC at the group level [10,11,12], and failed to do so at the individual level.

In the evaluation of patients with DOCs, however, the question is not to assess how but to assess whether the AC is tonotopically organized. In that respect, using source reconstruction algorithms in EEG may be superfluous because the main point is to detect small signal changes depending on the frequency encoding. These changes can be reflected on surface electrodes by variations of latency, amplitude and topography in response to sounds. To verify whether surface recordings could indirectly detect the presence of a frequency organization in the AC, we carried out a multiclass support vector machine (SVM) based classification of the individual EEG data recorded from 63 electrodes following the passive listening of various sounds in a group of 12 healthy volunteers and 2 patients with DOCs.

## Methods

The ethics committee of Besancon approved this study under N°11/605, and written informed consents were obtained from all participants or from their representatives.

### Participants

#### Healthy volunteers

Twelve healthy right-handed subjects (five males) aged between 26 and 36 years (mean: 29.8 ± 4.6) participated in this study. All reported normal hearing with no history of otological or neurological disorder.

#### Patients

Two unresponsive patients from a specialized unit were recruited in this study, none of them with a history of impaired auditory acuity.

Patient 1 was a 25-year old female diagnosed as UWS for 4 years following a cerebral anoxia sustained in a road-traffic accident. On the day of her EEG evaluation, patient 1 returned a diagnosis of low MCS with a Coma Recovery Scale–Revised (CRS-R) score of 8 (auditory function: localization to sound; visual function: startle; motor function: flexion withdrawal; oromotor/verbal function: oral reflexive movement; communication: none; arousal: eye opening without stimulation).

Patient 2 was a 37-year old female diagnosed as UWS for 4 years following a tonic–clonic seizure revealing a cerebral ischemia. On the day of her EEG assessment, the CRS-R diagnosis of patient 2 was UWS with a score of 4 (auditory function: startle; visual function: none; motor function: none; oromotor/verbal function: oral reflexive movement; communication: none; arousal: eye opening without stimulation).

### Experimental design

Auditory stimuli were tone bursts of 200 ms in duration, with 3 ms rise and fall time. Stimuli had four possible frequencies: 250, 500, 1000 and 2000 Hz and were presented in three stimulation conditions: either in the left ear, right ear, or in both ears simultaneously. As a consequence, there were 12 different types of stimuli. For each frequency, intensity was set to 70 dB SPL. A background pink noise of 50 dB SPL was simultaneously presented in order to mask possible extraneous noise during stimulation. The presentation of the stimuli was varied pseudo-randomly (successive repetition of the same type of stimulus was avoided) with interstimulus interval varying randomly between 600 and 800 ms.

A total of 2400 stimuli (200 of each type of stimulus) were played to each subject. Three short breaks of 5 minutes were presented during the experimental session. The experiment lasted for 45 minutes.

### EEG data acquisition and preprocessing

In the EEG experiment, all EEG channels were recorded using the OSG digital equipment (BrainRT, OSG bvba, Rumst, Belgium) with two Schwarzer AHNS epas 44 channels amplifiers (Natus, Munich, Germany). EEG signals were acquired from 64 electrodes at the positions of the 10/10 system using a 64 channel electrode cap (Easycap, EasycapGmbh, Ammersee, Germany). Sample frequency was set at 1000 Hz. Signal processing was performed using Cartool Software (http://brainmapping.unige.ch/Cartool.php). Epochs ranging from 50 ms prestimulus to 350 ms poststimulus were extracted for each experimental condition and participant. Signals were band-pass filtered using a second order Butterworth filter that eliminated the frequency interval 45–55 Hz that contains the power-supply artifacts. Other artifacts were subsequently eliminated by rejecting signals associated with electrodes that exhibited voltages exceeding ± 100 μV. In patients, an additional step was performed, consisting in visually removing trials with ocular artifacts. Data at artifact electrodes from each participant were interpolated using a 3-dimensional spline algorithm (average: 2% interpolated electrodes). The resulting signals were low-pass filtered at 30 Hz using an order two Butterworth filter. Finally, a baseline was defined for each electrode and stimulus using the average EEG voltage during the 50 ms preceding the stimulus onset and individual data were then recalculated against it.

### EEG data analysis

We carried out an individual EEG analysis following an identical protocol for both healthy volunteers as well as for patients with DOCs. The study had two main objectives:

1. Detection of a N100 wave. The N100 is a negative-going evoked potential peaking approximately around 100 ms after the onset of an auditory stimulus and expected to originate from the AC. The first goal was to investigate whether a N100 wave was generated by each healthy volunteer and each patient, in connection with the 12 different types of stimuli. Two different methods were implemented in order to assess the appearance of a N100 wave. First, the signal was visually inspected by a trained neuroscientist both at Cz and on the overlay of the event-related potential (ERP) waveforms for the 63 EEG channels. Next, in order to have a systematic and objective measure of the presence of the N100 wave, an automatic detection procedure was implemented based on the combination of a continuous wavelet transformation (CWT) and a Student-t test. More specifically, a t-CWT test [13] was used on the signals coming from Cz by first computing the Mexican Hat wavelet decomposition (with 120 scale parameters equidistributed from 8.33 to 250) of each trial corresponding to each stimulation frequency and modality (left ear, right ear, binaural). An average scalogram was then computed for each stimulation frequency and modality. The statistical significance of each point in these average scalograms was determined using the corresponding Student-t statistics. The N100 wave was determined by the minimum of this statistic in the interval [80ms, 150ms] poststimulus. Finally, the statistical significance of this minimum was assessed using a multivariate T²-Hotelling test that was performed at that point. The significance threshold was set to 5% (one-sided).
2. Detection of the frequency organization. For each individual, an automatic classification of the EEG signals was carried out corresponding to each frequency and stimulation scheme (left ear, right ear, binaural). For each trial, the 350 ms poststimulus signal from 63 EEG channels was used in the study. The multiclass classification was implemented using a SVM together with a one-versus-one strategy. The classification was carried out using Matlab's fitecoc function with default parameter values. This SVM binary classifier uses a linear kernel and, in order to provide a solution, uses a Sequential Minimal Optimization algorithm with a tolerance for the gradient difference between upper and lower violators of 10^-3^.The data were split into two disjoint parts for the classification: the training and the testing sets. The first one was used to estimate the parameters of the classifier, and the second allowed the evaluation of the classification performance. The global classification accuracy was determined using the following cross-validation scheme: the trials were randomly permuted and split into four groups containing the same number of elements. Each of these four groups was taken as a testing set for the classification, while the remaining trials were used for the training set; this operation yielded a mean computed using the four different accuracies corresponding to the four different choices of testing sets. The global average accuracy rate was obtained repeating 1000 times this operation. Furthermore, a permutation test was used to evaluate the statistical significance of the classification. Two kinds of performance were reported in connection with this study: first, the global accuracy associated to the stimulation modality, that is, the percentage of trials that was well classified with respect to the form of stimulation for all the frequencies; second, the accuracy of the classification of the sound frequencies for each form of stimulation (left ear, right ear, binaural).

## Results

**Fig 1:**
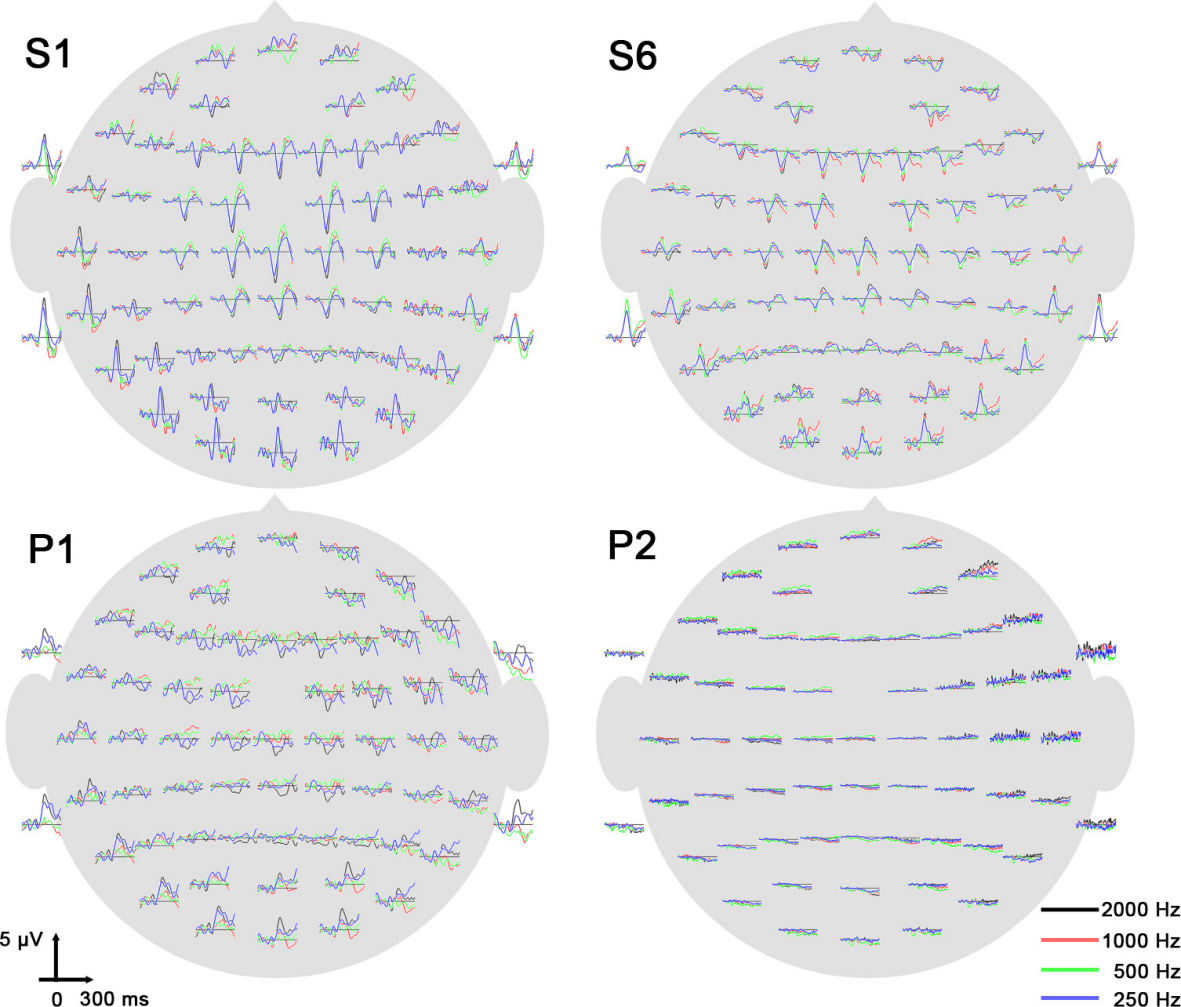
Signal changes in response to binaural stimulations in two healthy volunteers and the two patients. Around 100 ms, a negative wave was clearly noticeable over fronto-central electrodes and a positive wave over the temporal electrodes for all stimulation frequencies in the two healthy subjects. This response was present but less marked in patient 1. In patient 2, no N100 was visually detected, but the automatic procedure statistically detected specific responses despite the small amplitude of the signal.

An example of the EEG signal recorded over the 63 electrodes is shown in Fig 1.

### Individual measures in healthy volunteers

**Fig 2:**
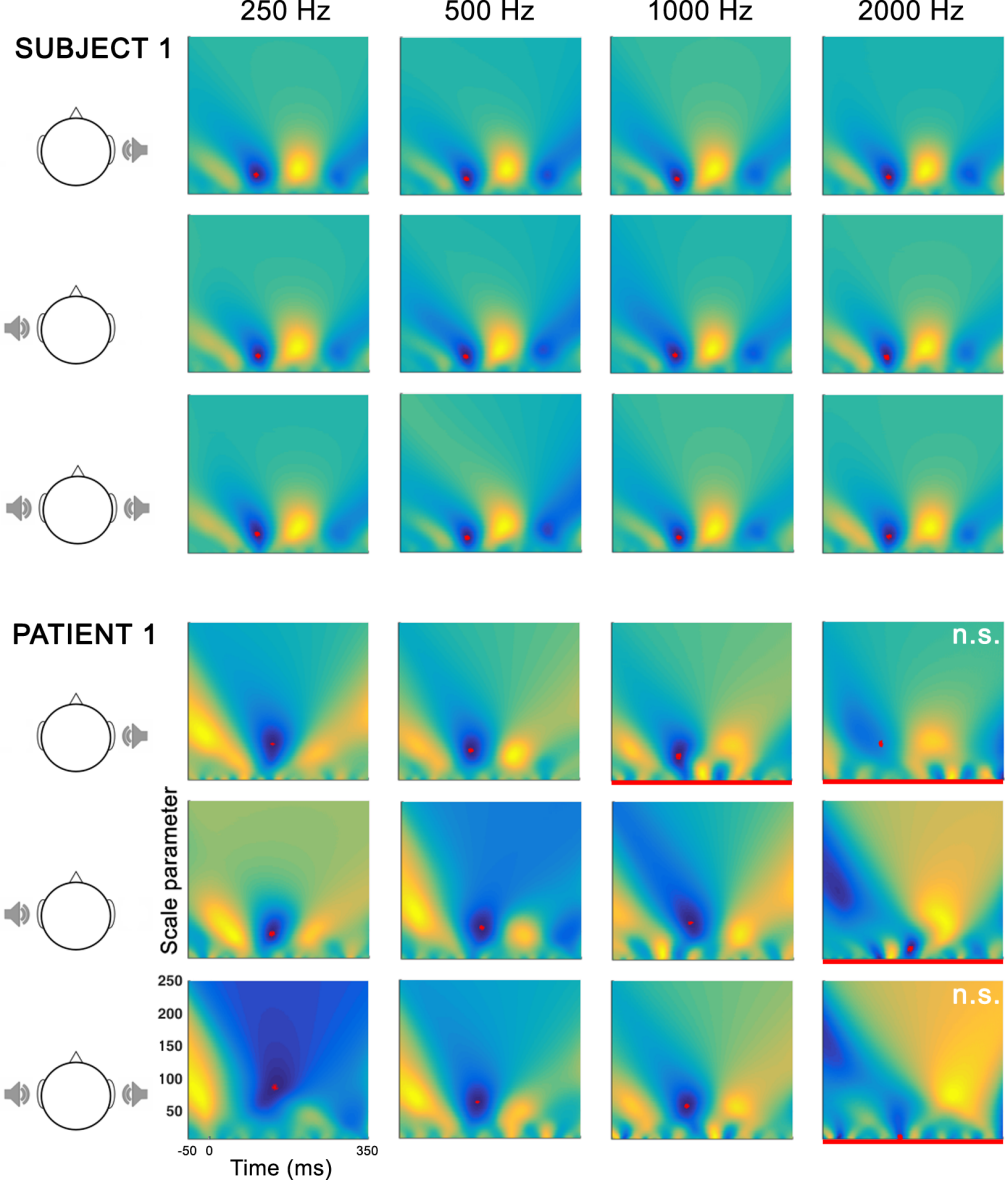
Example of automatic detection procedure of the N100 in a healthy subject and in a patient. Each figure represents the average normalized scalogram for each frequency and stimulation modality. The blue color indicates negative values and the red dots represent the minimum of the average normalized scalogram in the interval [80ms, 150ms] poststimulus. In patient 1, the N100 was not visually detected under four stimulation conditions (indicated with a red line).

The visual and automatic procedures easily detected (p-value < 0.01 for the t-CWT method) a N100 wave for all frequencies and stimulation conditions for almost all healthy subjects (see Fig 2 for an example). Only for subject 2, whose N100 response was globally of lower amplitude than for the other participants, the automatic procedure could not detect a N100 at 250 Hz after left ear stimulation and at 2000 Hz after binaural stimulation. Moreover, in this subject, no N100 was visually identifiable at 500 Hz after binaural stimulation and at 2000 Hz after right ear stimulation.

The SVM setup was able to globally classify all the frequencies in all healthy volunteers (see Table 1) following binaural stimulation. After monaural stimulation, a correct classification was obtained in all subjects but one (subject 4). Three subjects (subject 1, 5 and 6) had all frequencies correctly classified for at least one out of the three stimulation conditions. The N100 resulting from 250 Hz and 2000 Hz stimulations were the most accurately classified, regardless of the ear that had been stimulated.

**Table 1:** Results of the individual frequency and stimulation modality classifications for the 12 healthy subjects and for the 2 patients.

		Right ear stimulation					Left ear stimulation					Binaural stimulation				
		All	250 Hz	500 Hz	1000 Hz	2000 Hz	All	250 Hz	500 Hz	1000 Hz	2000 Hz	All	250 Hz	500 Hz	1000 Hz	2000 Hz
**subject 1**	*accuracy*	0,390155	0,450677	0,372679	0,371035	0,371755	0,378133	0,443268	0,32407	0,324217	0,425543	0,356697	0,384373	0,348419	0,332431	0,36847
	*p-value*	**0**	**0**	**0**	**0**	**0**	**0**	**0**	0,012	0,021	**0**	**0**	**0**	0,003	0,012	**0**
**subject 2**	*accuracy*	0,325263	0,356764	0,310273	0,303522	0,335345	0,29917	0,272382	0,267126	0,30116	0,360297	0,336327	0,353536	0,32182	0,304015	0,371129
	*p-value*	**0**	**0,001**	0,026	0,066	**0,006**	**0,003**	0,238	0,326	0,056	**0**	**0**	**0,001**	**0,011**	**0,049**	**0**
**subject 3**	*accuracy*	0,307513	0,3173	0,287372	0,284653	0,34532	0,304816	0,333912	0,295269	0,269588	0,324768	0,317719	0,332521	0,308656	0,297162	0,33753
	*p-value*	**0**	0,018	0,113	0,126	**0,002**	**0,001**	**0,003**	0,076	0,266	0,011	**0**	**0,005**	0,032	0,081	**0,004**
**subject 4**	*accuracy*	0,279314	0,275093	0,299844	0,271217	0,275903	0,26867	0,267158	0,277831	0,289088	0,244815	0,316715	0,352695	0,292592	0,288461	0,338206
	*p-value*	0,052	0,188	0,081	0,294	0,177	0,128	0,288	0,227	0,117	0,525	**0**	**0**	0,131	0,123	**0,007**
**subject 5**	*accuracy*	0,346827	0,357691	0,378376	0,331369	0,324407	0,403167	0,43117	0,372186	0,383236	0,431316	0,343682	0,379573	0,303513	0,339966	0,355757
	*p-value*	**0**	**0,006**	**0**	**0,006**	**0,005**	**0**	**0**	**0**	**0**	**0**	**0**	**0**	0,027	**0,008**	**0**
**subject 6**	*accuracy*	0,440448	0,504192	0,38847	0,398513	0,475498	0,375278	0,427844	0,317185	0,3639	0,397584	0,379294	0,416429	0,377026	0,339987	0,388456
	*p-value*	**0**	**0**	**0**	**0**	**0**	**0**	**0**	0,027	**0,001**	**0**	**0**	**0**	**0**	**0,004**	**0**
**subject 7**	*accuracy*	0,321306	0,328015	0,313598	0,324449	0,324654	0,316479	0,342468	0,311793	0,297896	0,318844	0,304933	0,293009	0,292844	0,311515	0,327546
	*p-value*	**0**	**0,008**	0,033	0,022	0,018	**0**	**0,002**	0,025	0,103	0,015	**0,001**	0,111	0,083	0,036	0,014
**subject 8**	*accuracy*	0,33689	0,374635	0,316184	0,345789	0,315337	0,306231	0,349529	0,261826	0,302632	0,315833	0,290845	0,30365	0,27041	0,314174	0,279173
	*p-value*	**0**	**0**	0,024	**0,001**	0,021	**0**	**0,001**	0,375	0,058	0,031	**0,009**	0,071	0,275	0,023	0,187
**subject 9**	*accuracy*	0,360722	0,418192	0,334549	0,317916	0,377759	0,354852	0,398909	0,341471	0,32809	0,355688	0,352613	0,378011	0,30365	0,343212	0,390224
	*p-value*	**0**	**0**	**0,007**	0,016	**0**	**0**	**0**	**0,003**	0,013	**0**	**0**	**0**	0,048	**0,002**	**0**
**subject 10**	*accuracy*	0,342552	0,358983	0,311838	0,328712	0,375475	0,326622	0,34608	0,309251	0,296837	0,358546	0,358657	0,383319	0,331397	0,288334	0,435737
	*p-value*	**0**	**0**	0,025	**0,007**	**0**	**0**	**0,002**	0,038	0,074	**0**	**0**	**0**	**0,009**	0,099	**0**
**subject 11**	*accuracy*	0,314137	0,359943	0,277537	0,343772	0,280159	0,362976	0,427391	0,366838	0,319447	0,343717	0,315447	0,383212	0,30588	0,277158	0,30082
	*p-value*	**0**	**0**	0,186	**0,005**	0,175	**0**	**0**	**0**	0,025	**0,002**	**0**	**0**	0,053	0,21	0,062
**subject 12**	*accuracy*	0,336366	0,344635	0,31125	0,332638	0,360772	0,313538	0,353983	0,281896	0,33893	0,284313	0,320983	0,366253	0,288482	0,308026	0,325682
	*p-value*	**0**	**0,008**	0,027	**0,004**	**0,003**	**0**	**0,001**	0,138	0,011	0,187	**0**	**0,002**	0,092	0,045	0,013
**patient 1**	*accuracy*	0,260742	0,299086	0,244047	0,215966	0,287146	0,307839	0,294642	0,314267	0,316457	0,31063	0,314117	0,344067	0,350404	0,292108	0,272593
	*p-value*	0,243	0,093	0,504	0,839	0,138	**0**	0,132	0,025	0,04	0,034	**0**	**0,004**	**0,003**	0,131	0,218
**patient 2**	*accuracy*	0,262713	0,242158	0,249192	0,297457	0,270851	0,243922	0,244665	0,250506	0,237704	0,250699	0,258795	0,261171	0,253373	0,295188	0,23263
	*p-value*	0,311	0,829	0,491	0,258	0,057	0,598	0,74	0,358	0,461	0,555	0,326	0,253	0,707	0,156	0,576

The p values in bold correspond to accuracies that are significantly different from the accuracy associated to a random distribution of the classification labels (25%) according to a permutation test at a significance level of 1%.

### Individual measures in patients

In patient 1, the automatic procedure detected a N100 at all frequencies except for 2000 Hz after right ear and binaural stimulation. The visual method detected a N100 at 250 Hz and 500 Hz for all stimulation conditions and at 1000 Hz for left ear and binaural stimulations. No N100 was visually detected at 2000 Hz (Fig 2). The SVM classification detected a global frequency organization after monaural left and binaural stimulations (p< 0.0001 for both). After binaural stimulation, a specific frequency organization was observed for 250 and 500 Hz tones. Despite the visual and/or automatic detection of a N100 at some specific frequencies, the classification procedure could not detect any frequency organization after right ear stimulation (Table 1).

In patient 2, no N100 wave was visually detected. The automatic detection procedure identified a N100 only after binaural stimulation at 1000 Hz. In this patient, no correct classification was achieved for any of the frequencies and stimulation conditions (see Table 1).

## Discussion

In this study, we found that a multiclass SVM based classification successfully detected signal differences in the encoding of frequencies at the individual level. In all healthy subjects, a frequency-selective processing was evidenced after binaural stimulation and in all but one after left ear or right ear. When this method was applied at the bedside of two patients with DOCs, large discrepancies were found, putting the emphasis on their specific cortical organization. Patient 1 could accurately discriminate frequencies after left ear and binaural stimulations but not after right ear stimulation, suggesting a possible damage of the left AC. Unlike patient 1, no successful classification could be obtained for patient 2, even though a N100 was detected at 1000 Hz for binaural stimulation. In this patient, there is strong evidence of an absence of residual organization in her AC. These results show that the classification method presented here is helpful to indirectly measure the integrity of the AC in both ears at the individual level using EEG signals.

In the hierarchical evaluation of awareness in patients with DOCs, the approach presented here is an essential step to understand whether sound perception is preserved in these patients, and essentially provides an additional information to the question frequently asked by relatives of patients with DOCs: “can he/she hear?”. In patient 1, the partial integrity of the AC revealed by SVM analysis encourages subsequent investigations of higher cognitive functions such as speech comprehension, especially by right ear stimulation. In patient 2, a precise measurement of the auditory brainstem responses at all stimulation frequencies is mandatory to understand which steps of the acoustic processing are damaged.

In general, most EEG investigations of the integrity of the AC in patients with DOCs have only focused on the presence of a N100 wave, or a related component named mismatch negativity, in patients. Previous studies have shown that the absence of the N100 was a good predictor of bad outcome[14,15], but there was a controversy on whether its absence could actually predict recovery[14,15,16,17]. Here, results from patient 2 suggest a dissociation between the presence of a N100 and the presence of a frequency organization. Tonotopic studies on the N100 suggest that this wave may be generated from tonotopic but also from non tonotopic areas [18]. This may explain this distinction between a response and a frequency organization of the AC and why no correlation was found between the presence of a N100 and the transition from coma/UWS to MCS [19]. Future investigations taking into account the frequency encoding are necessary to reveal whether this could improve the prognosis of these patients. Other methods based on the investigation of the mismatch negativity fail to really measure the integrity of the AC because the processes recorded with the mismatch negativity include automatic attention and memory, and are consequently more complex than the frequency-selective analysis of the AC.

The approach described here presents several advantages. The patient does not need to be awake during the experiment because a response from the AC can be obtained in various situations including sleep [20] or sedation [5]. Furthermore, the passive stimulations used in this study can be divided in several smaller sessions and adapted to the patient. It is also important to note that the classification method used in the present study did not only take into account fronto-central and temporal electrodes, known to be the regions where the N100 is the most prominent, but the whole set of 63 electrodes in the whole time period. Such methodology is adapted to patients with cerebral damage whose ERP may be distributed over different scalp areas than healthy subjects.

If the present results can serve as a ground for future investigations, one cannot also fully exclude that other factors might have impacted the classification. A first one is the stimulus intensity. Since it is not possible to precisely measure the individual hearing threshold of patients with DOCs, loudness changes may have impacted the latency and the classification of sounds. This problem, recurrent in all auditory experiments, can nevertheless hardly explain the classification observed in patient 1, especially because of the clear detection of a N100 in some stimulation conditions. Furthermore, some stimulation frequencies were less correctly classified than others in almost all subjects. For example, revealing differences of signal between 500 and 1000 Hz was often difficult. For these reasons, an absence of correct classification in patients with DOCs at some or all frequencies always has to be considered with care. Negative findings in electrophysiological and neuroimaging protocols used to detect residual cognition in patients with DOCs should never be taken as evidence of lack of awareness, but rather that the method was not sensitive enough at the time of the recording [21]. This is all the more true that brain injuries can also significantly alter the characteristics (latency, localization, shape) of the electrophysiological signal. Furthermore, impairment in arousal regulation is frequently observed in these patients because of damages to thalamocortical structures [22]. The variations of arousal and the inconsistent evidence of awareness indicate that the same protocol should be repeated over several sessions to confirm the first results.

Another theoretical issue is the absence of a real understanding of what is ‘behind’ the SVM classification. First, even though electrophysiological differences were found on the surface activity, suggesting the presence of a tonotopic organization, no information is given about the structure of this tonotopic organization within the AC. Second, the analysis was performed on a large 350 ms time window. Cerebral processes other than the N100 might have contributed to the classification. Third, recording and analyzing ERPs on the whole scalp means that regions outside the AC may have been responsible for the accuracy of the SVM classification. However, these limitations do not exclude that sounds are encoded differently according to their frequency and that frequency processing is preserved, which was our main objective. To verify this, EEG could be used as a first line assessment and doubtful results could be explored further by fMRI of high spatial resolution [7].

Although the sample of tested patients is small, these initial results provide evidence that SVM classification on surface electrodes can be a powerful method to indirectly measure the frequency-selective processing of the AC at the patient’s bedside. In that respect, this method can help define an accurate diagnosis of awareness in patients with DOCs by enriching the hierarchy of cognitive tasks which will perform the assessment of the residual cognitive functions of these patients. In the hierarchical evaluation of residual auditory cognition, this method fulfills the missing link between the evaluation of acoustic processing and more complex perceptual and phonological processes. Should the functional organization of the primary AC be preserved, more elaborate neuroimaging investigations of the AC focusing on speech perception, speech comprehension and semantic processing would then be possible. This method may also have a prognostic value in the recovery of consciousness. Whereas investigations of the N100 alone have brought contradictory information on whether its presence predicts recovery of comatose patients, our results suggest that the N100 alone does not sufficiently report the integrity of the AC. The presence of a preserved frequency processing may thus have a more accurate prognosis value of recovery. However, the replication of the method on a larger sample of patients with DOCs is necessary to confirm its diagnosis and prognosis value. It is also important to note that such an approach which is able to detect frequency processing at the individual level can be extended to other pathological populations. For example, several studies performed at the group level have suggested that the AC of otosclerotic patients was tonotopically disorganized, and that a normal organization is recovered after corrective surgery [23]. Although our approach cannot give exact information about the tonotopic organization within the AC, a recovered frequency organization could be confirmed at the individual level by comparing the classification accuracy before and after surgery. A disturbed tonotopic organization has also been found in patients with schizophrenia [24]. Our approach could be help to assess the level of disturbance at the individual level.
